# How to Use Plastic Fillings

**Published:** 1874-10

**Authors:** 


*How to Use Plastic Fillings.*—Thos. Fletcher, F. C. S., writes as follows upon this subject:

"Of all conditions necessary for a filling which hardens after the operator leaves it, none is more imperative than *the absolute dryness of the cavity.* Neglect of this entails certain failure in every case as regards the production of a really perfect plug. With gold or tin, which can be wedged, and which are hard and safe the moment they are left, the absolute exclusion of moisture is of little importance in comparison; but with a plastic filling it is the necessary condition above all others; and I believe from experience that the neglect of this vital point causes the failure of more amalgam plugs than all other causes of every kind put together. When an amalgam plug is inserted in a cavity containing moisture, a trace is always left between the plug and the tooth substance which no amount of force will drive out. When the pressure of the instrument is removed the moisture gradually forces its way back until it forms a layer all over the cavity, the force of capillary action being quite sufficient to alter the form of hardest amalgam before it is set, and to produce a space between the plug and tooth which may be distinctly felt with a fine probe in a few hours or days. The action of moisture may be rendered distinctly visible by packing a plug in a wet cavity of glass, covered with a colored solution, and watching the result. If the cavity is perfectly dry the moisture has no power to penetrate between the plug and the tooth, unless the plug shrinks or is disturbed whilst soft.

In cavities difficult to keep dry the best safeguard is the free use (after drying as perfectly as possible) of a solution of gum copal in ether or resin in carbolic acid. The latter not only hardens the dentine but offers very great resistance to the passage of moisture. Prepare the cavity, dry it and pack firmly in a plug of amadou soaked in the resin and carbolic acid solution, leaving it in whilst the amalgam is prepared. Then remove the plug, wipe out the cavity with a similar one, and afterwards with dry amadou. If the amalgam can be got into contact with the walls of the cavity before moisture penetrates, it is safe; if not, it is better to try again rather than risk eventual failure. If this treatment is carried out and an amalgam is used which is free from shrinkage, the line joining the plug and tooth is inappreciable to the finest probe months or years afterwards; if the moisture has obtained entrance, the division can be felt in a day or two at most—generally in a few hours. An operator who forgets this or overlooks its importance damages his own reputation and the reputation of amalgams also, without a just cause. An amalgam properly made and properly worked is absolutely safe as a permanent filling; and the operator may blame his own neglect for every failure."

---

